# PD/1-PD-Ls Checkpoint: Insight on the Potential Role of NK Cells

**DOI:** 10.3389/fimmu.2019.01242

**Published:** 2019-06-04

**Authors:** Silvia Pesce, Marco Greppi, Francesco Grossi, Genny Del Zotto, Lorenzo Moretta, Simona Sivori, Carlo Genova, Emanuela Marcenaro

**Affiliations:** ^1^Department of Experimental Medicine, University of Genoa, Genoa, Italy; ^2^Centre of Excellence for Biomedical Research, University of Genoa, Genoa, Italy; ^3^Medical Oncology Unit, Fondazione IRCCS Ca' Granda Ospedale Maggiore Policlinico, Milan, Italy; ^4^IRCCS, Istituto Giannina Gaslini, Genoa, Italy; ^5^Immunology Unit, IRCCS Ospedale Bambino Gesù, Rome, Italy; ^6^Lung Cancer Unit, IRCCS Ospedale Policlinico San Martino, Genoa, Italy

**Keywords:** NK cells, PD-1, PD-L, KIR, NKG2A, immune checkpoint, immune checkpoint blockade, immunotherapy

## Abstract

The identification of inhibitory NK cell receptors specific for HLA-I molecules (KIRs and NKG2A) provided the molecular basis for clarifying the mechanism by which NK cells kill transformed cells while sparing normal cells. The direct interactions between inhibitory NK cell receptors and their HLA-I ligands enable NK cells to distinguish healthy from transformed cells, which frequently show an altered expression of HLA-I molecules. Indeed, NK cells can kill cancer cells that have lost, or under express, HLA-I molecules, but not cells maintaining their expression. In this last case, it is possible to use anti-KIR or anti-NKG2A monoclonal antibodies to block the inhibitory signals generated by these receptors and to restore the anti-tumor NK cell activity. These treatments fall within the context of the new immunotherapeutic strategies known as “immune checkpoint blockade.” These antibodies are currently used in clinical trials in the treatment of both hematological and solid tumors. However, a more complex scenario has recently emerged. For example, NK cells can also express additional immune checkpoints, including PD-1, that was originally described on T lymphocytes, and whose ligands (PD-Ls) are usually overexpressed on tumor cells. Thus, it appears that the activation of NK cells and their potentially harmful effector functions are under the control of different immune checkpoints and their simultaneous expression could provide additional levels of suppression to anti-tumor NK cell responses. This review is focused on PD-1 immune checkpoint in NK cells, its potential role in immunosuppression, and the therapeutic strategies to recover NK cell cytotoxicity and anti-tumor effect.

## Introduction

The immune system defends our body against foreign microbes/antigens, while simultaneously preventing self-reactivity. To this aim, immune cells are regulated by a balance between inhibitory and activating receptors/co-receptors expressed on their surface, which, upon interactions with their ligands, deliver negative, or positive signals that dictate the outcome of immune responses.

Receptors delivering inhibitory signals function as immune checkpoints and play a more general role in maintaining peripheral tolerance and preventing autoimmunity (1, 2).

However, immune-regulation displays also its negative tradeoffs. Thus, local tolerance in the tumor microenvironment represents a common survival strategy, exploited by different tumors to escape elimination by the immune system (3).

Immune checkpoint blockade is based on the use of monoclonal antibodies (mAbs) directed against inhibitory checkpoints expressed by immune cells (or their ligands expressed by tumor cells). The mAb-mediated disruption of these receptor/ligand interactions may revert the functional inhibition of these cells and restore an effective anti-tumor cytotoxic activity, possibly leading to durable tumor regression.

The best-known immune checkpoints are represented by CTLA-4 and the axis including PD-1 and its ligands PD-Ls (PD-L1/PD-L2). Both CTLA-4 and PD-1 were originally identified in T lymphocytes, while PD-Ls may be expressed on different tumor cells. Since the discovery of these immune checkpoints, the innovative cancer immunotherapeutic approach was focused on the restoration of T cell-mediated specific responses to tumor antigens.

Anti-CTLA-4 mAbs, such as ipilimumab, were the first of this class of immunotherapeutics that became available in clinical practice achieving the US FDA approval for the treatment of metastatic melanoma (4, 5).

The first PD-1/PD-Ls disrupting agent approved for the treatment of solid tumors was the anti-PD-1 nivolumab; subsequently, several PD-1/PD-Ls blockers have been introduced in clinical practice and many others are currently under investigations across different solid and hematologic malignancies, including non-small cell lung cancer (NSCLC), melanoma, head&neck cancer, renal cell carcinoma and urothelial carcinoma, and high-grade Hodgkin's lymphoma. In addition, various combination therapies have recently been explored in clinical trials, and some combination therapies are being introduced in clinical practice, with particular reference to NSCLC, including combinations of nivolumab and ipilimumab, or platinum-based chemotherapy in association with the anti-PD-1 pembrolizumab, or a combination of platinum-based chemotherapy plus the anti-PD-L1 atezolizumab and the anti-angiogenic bevacizumab. It is possible that combination therapies may earn a progressively prominent role in clinical practice in the upcoming years (6, 7). More recently, maintenance with the anti-PD-L1 agent durvalumab after chemo-radiation for unresectable, locally advanced NSCLC, resulted in a significant improvement in terms of progression-free survival and overall survival (8). While, on one hand, immunotherapy with immune checkpoint inhibitors is characterized by impressive results across various solid and hematologic tumors, on the other hand, the great variability of response among patients (indeed a still large fraction of patients fails to respond), suggests that the complex biology of immune checkpoint pathways has not yet been fully understood. Notably, the documented expression of PD-L1 on tumors might influence the clinical decision of treating patients with immune checkpoint inhibitors in specific settings; indeed, strong expression of PD-L1 (≥50%) is required for prescribing pembrolizumab as first-line treatment for advanced NSCLC; similarly, a positive expression of PD-L1 (at least 1%) is necessary for the administration of pembrolizumab in second and further lines and for the administration of durvalumab after chemo-radiation with curative intent for locally advanced NSCLC. However, the available tumor samples are frequently represented by biopsies that may yield inadequate information on the actual PD-L1 expression, eventually representing a potential issue in the management of NSCLC (9). Furthermore, the predictive role of PD-L1 expression has been questioned by additional clinical data; in first place, the outcomes of PD-1/PD-L1 inhibitors in other malignancies apart from NSCLC do not appear to be influenced by PD-L1 expression. Furthermore, in NSCLC, single-agent treatments with nivolumab and atezolizumab for previously treated disease were effective regardless of PD-L1 expression. Similarly, the outcomes achieved by combination regimens involving multiple immune checkpoint blocking agents, such as ipilimumab plus nivolumab in solid tumors, including renal cell cancer (RCC), NSCLC, and melanoma, or involving platinum-based chemotherapy plus a PD-1 or PD-L1 inhibitor in NSCLC were not influenced by PD-L1 expression (10–13). Taken together, these data suggest that other factors, apart from the mere percentage of tumor cells expressing PD-L1, must play a relevant role in immune checkpoint blockade (14).

In this context, independent research groups published data on the contributions of host cells in the PD-1/PD-L1 blockade mediated cancer immunotherapy in patients with PD-L1-negative tumors responding to this blockade therapy (15–18).

Indeed, besides tumor cells, various types of host cells also constitutively express PD-L1, or can upregulate its expression upon stimulation with inflammatory cytokines, including IFN-gamma. These observations imply that PD-L1 from tumor and/or host compartment works in concert to dampen the anti-tumor immune response (19). In addition to the PD-1/PD-L1 axis, also the PD-1/PD-L2 interaction may play an important role in evading anti-tumor immunity, suggesting that PD-1/PD-L2 blockade must be considered for optimal immunotherapy in PD-L2-expressing cancers, such as RCC and NSCLC (20, 21).

### The Multifaceted Nature of NK Cells

NK cells are classified as lymphocytes on the basis of their origin from the common lymphoid progenitor cell in the bone marrow. However, different from T and B lymphocytes, they do not express antigen-specific cell surface receptors encoded by rearranging genes. Thus, NK cells are considered to be players of innate immune defenses, and, in particular, represent cytotoxic innate lymphoid cells (ILCs) (22, 23). They were originally identified over 40 years ago (24, 25), on the basis of their ability to kill tumor/virus-infected cells in the absence of prior activation. Later, NK cells have been recognized as immunoregulatory cells, secreting pro-inflammatory cytokines, and many chemokines, and expressing different receptors for both cytokines and chemokines. This means that NK cells may recruit and may be recruited to inflammatory sites where they can colocalize with other immune cells, including dendritic cells with which NK cells can cooperate (22, 26, 27). These different cell-to-cell interactions endow NK cells with regulatory function affecting both the quality and the strength of adaptive immune defenses (23, 28).

Several lines of evidence indicate that NK cells or their receptors play a critical role in immunosurveillance of spontaneous tumors and in preventing tumor metastases. In addition, it has been shown that impairment in NK cytotoxic activity is associated with a higher cancer risk (29). Indeed, tumors have evolved mechanisms to escape NK cell control. This knowledge prompted efforts to exploit NK cell functions for better management of cancer patients.

### The Discovery of NK Cell Immune Checkpoints

Similar to T cells, NK cells express surface receptors that can be targeted in checkpoint blockade strategies (30, 31).

The first NK cell immune checkpoints were identified by Alessandro Moretta in 1990 with the discovery of p58 molecules that were later called killer cell immunoglobulin-like receptors (KIRs). He demonstrated that KIRs were specific for allotypic determinants of HLA-I molecules. He also greatly contributed to the identification of additional receptors interacting with HLA-I molecules including the HLA-E specific CD94/NKG2A heterodimer (32–34). These findings represented a true revolution in the field of human NK cell biology and opened new avenues in the field of NK cell-based immunotherapeutic approaches.

Initially, NK cell-based immunotherapy had mainly been exploited to treat hematological malignancies and relied either on the adoptive transfer of NK cells or on NK cells generated from transplanted hematopoietic stem cell to treat high-risk leukemia (35, 36).

Recent approaches have been based on mAb-mediated blockade of specific NK cell immune checkpoints (37).

In 1999, Alessandro Moretta, together with Eric Vivier, Hervé Brailly, Marc Bonneville, Jean-Jacques Fournié and Francois Romagné, founded Innate Pharma, the first Biotech Company that aimed to target NK cells in innovative anti-tumor immunotherapeutic strategies (https://www.innate-pharma.com/en/profile/founders). Starting from mAbs generated in Alessandro's lab, directed against the HLA-I specific inhibitory receptors, the first two immune checkpoint inhibitors were generated: lirilumab targeting pan-KIR2D and monalizumab targeting NKG2A. These therapeutic antibodies, with the capacity to disrupt the interactions between KIR or NKG2A and HLA-I, are expected to unleash the anti-tumor NK cell cytotoxic activity mimicking “missing-self” response. Both lirilumab and monalizumab have been shown to be safe with limited side effects upon prolonged treatments in phase I clinical trials (38). These agents are currently undergoing phase I/II clinical trials across a range of hematologic and solid tumors in monotherapy or in combination with other agents, including rituximab (an anti-CD20 mAb), and other forms of immune checkpoint blockade (39–41).

In addition to KIR and NKG2A, other inhibitory checkpoints may be expressed on NK cells. They include the T-cell Ig and ITIM domain (TIGIT), CD96 (TACTILE), LAG-3 and TIM-3.

TIGIT and CD96 are co-inhibitory receptors expressed on both T and NK cells and compete with the activating receptor DNAM-1 for binding to PVR (CD155) and Nectin-2 (CD112) (42). TIGIT expression has been reported as upregulated in tumor-associated NK cells in different malignancies (43). Thus, it has been hypothesized that TIGIT could play a role in carcinogenesis due to its ability to inhibit NK cell cytotoxicity. A recent study provided evidence that TIGIT blockade may induce anti-tumor immune activity in preclinical models, and its combination with PD-1/PD-L1 inhibitors is being explored (44). Pre-clinical data also showed that blockade of CD96 alone or in combination with anti-PD-1 or anti-CTLA-4 or doxorubicin promotes NK cell activity (in terms of IFN-γ release) and a better control of tumor progression (45, 46).

LAG-3 is a negative costimulatory receptor homologous to CD4 and expressed on activated T and NK cells (47). High-affinity ligands for LAG-3 are HLA-II molecules that are mainly expressed by antigen presenting cells, but also by some cancer cells. Despite its inhibitory activity has been defined only in T cells, this immune checkpoint is currently considered as a good target for immunotherapy because of its potential to activate both T and NK cells. Indeed, LAG-3 mAbs are currently in pre-clinical development in association with standard chemotherapy (NCT02614833) and in combination with anti-PD-1 therapy (NCT02676869, NCT01968109).

TIM-3 is a checkpoint receptor that binds several ligands including galectin-9 (Gal-9) (48), phosphatidylserine on apoptotic cells (49), high mobility group box 1 (HMGB1) (50), and CEA-related cell adhesion molecule-1(CEACAM1) (51). TIM-3 is expressed on both adaptive and innate immune cells (52, 53). The engagement of this inhibitory checkpoint on NK cells may have different and opposite effects (53). These divergent functions are likely associated with the existence of multiple and different TIM-3 ligands. Blockade of TIM-3 could restore T-cell effector function in preclinical models and result in increased NK cytotoxicity (54).

### The Identification of the PD-1^+^ NK Cell Subset

The effect of the PD-1/PD-L1 blockade has been usually attributed to the restoration of cytotoxic T lymphocyte activity, and killing of tumor cells expressing HLA-I molecules. However, a partial or complete loss of HLA-I expression is one of the most frequent mechanisms of tumor escape from the host's immune system in different human tumor types. In this context, it is important to remember the “missing self” hypothesis postulated by Karre in 1986 (55), and formally proven in humans by Alessandro Moretta (56–59). The “missing self” hypothesis stated that absence, or reduced expression, of *self*-HLA-I molecules may be sufficient to render a target cell susceptible to killing by NK cells. Thus, NK cells recognize tumors that avoid T cell-mediated killing through abnormal or absent HLA-I expression. This is clinically relevant for patients with tumors displaying low levels of HLA-I at diagnosis and suggests the potential of NK cell-based adoptive immunotherapy.

**Figure 1:**
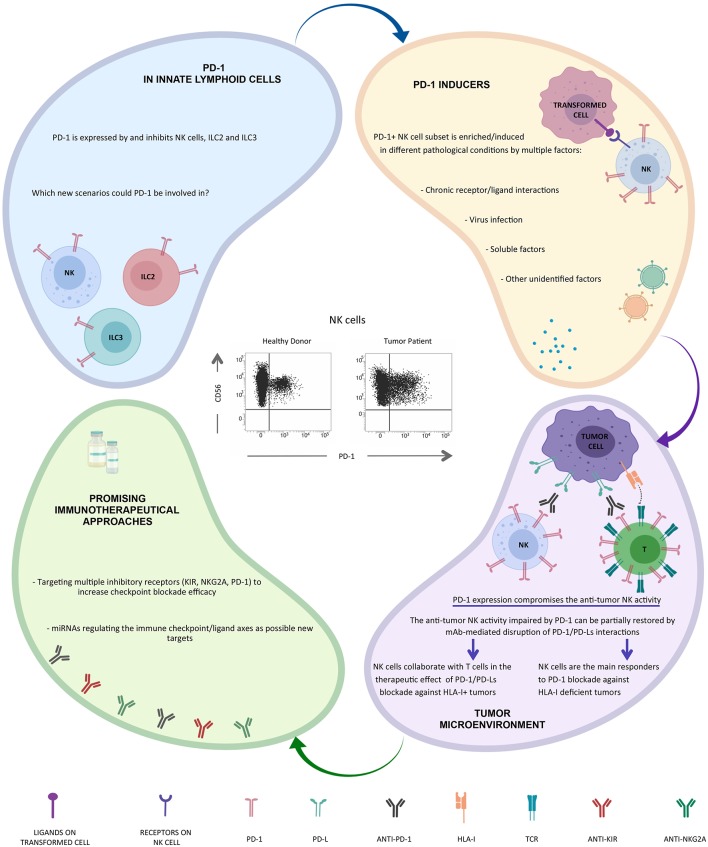
New insight on the potential role of PD-1 in NK cells and in other ILCs.

Along this line, we were convinced that even NK cells could contribute to the clinical benefit of immunotherapeutic strategies targeting PD-1/PD-L1 axis, but to confirm our hypothesis we first had to demonstrate that even NK cells could express PD-1 (Figure 1).

Initially, we tested different mAbs to check the best reagent to analyze this receptor on NK cells. We selected a reagent generated by our friend and international scientist Daniel Olive, as the most performing among all the tested mAbs (60, 61). Regarding the biological samples, we started the analysis of healthy donors, and thanks to the high number of healthy donors analyzed and also to the excellent reagent used, we were able to identify a subset of NK cells expressing high level of PD-1 in 25% of the donors analyzed (62). In order to verify what we were observing, we increased the cases and the controls; this allowed us not only to confirm this expression but also to characterize this new PD-1^+^ NK cell subset. PD-1 expression was confined to CD56^dim^ NK cells and, if present, to CD56^neg^ NK cells, whereas the CD56^bright^ subset was consistently PD-1^neg^. By comparing the PD-1^+^ and PD-1^neg^ NK cells derived from the same healthy donor, we found that the PD-1^+^ subset was confined to fully mature NK cells. Indeed, these PD-1^+^ NK cells were homogeneously characterized by the CD56^dim^KIR^+^LIR-1^+^NKG2A^neg^CD57^+^NCR^dim^ phenotype.

The fact that only one fourth of the individuals analyzed were characterized by a PD-1^+^ NK cell subset could be the result of given acute or chronic infection affecting only part of the population. Further analysis revealed that a direct correlation between HCMV infection and presence of a PD-1^+^ NK cell subset could be established. Indeed, the totality of PD-1^+^ individuals was seropositive for HCMV. Furthermore, as expected, the PD-1^+^ donors showed a reconfiguration of the NK cell receptor repertoire, typically induced by HCMV infection (62, 63). Interestingly, different PD-1 mRNA splicing isoforms and a cytoplasmic pool of PD-1 protein are detectable in virtually all NK cells analyzed (mainly CD56^dim^), thus indicating a possible rapid recruitment of this molecule on cell surface in response to precise, yet undefined, stimuli (64).

Once the presence of PD-1 on healthy donor NK cells was demonstrated, we moved to cancer patients. Given our previous expertise in ovarian cancer (65), we decided to focus our attention on NK cells derived from this kind of tumor patients. Our analyses showed that PD-1+ NK cells were detectable in the peripheral blood (PB) of the majority of these patients. More importantly, this NK cell subset was further increased in the tumor microenvironment, thus suggesting a possible accumulation/induction of this subset in this compartment. Again, the PD-1+ NK cells were confined to the CD56^dim^ NK subset, although our ongoing analyzes indicate that the features of the tumor-associated PD-1^+^ NK cell subset are different from those of the subset present in healthy donors (manuscript in preparation).

As the interest on PD-1^+^ NK cells is now turned on, several papers have been recently published, confirming the presence of the PD-1^+^ NK cell subset in tumor patients. In particular higher proportions of PD-1^+^ NK cells can be also detected on PB NK cells from multiple myeloma or Kaposi sarcoma patients and on PB and tumor-associated NK cells in head&neck cancer patients (41, 66). *In vitro*, PD-1 expression may be induced on NK cell surface upon persistent stimulation by tumor cells expressing ligands for activating NK receptors (66). In addition, virus infection (e.g., HCMV) (62) and/or soluble factors released in the tumor microenvironment (including endogenous glucocorticoids) may be involved in PD-1 induction (67, 68) (Figure 1).

This phenotype correlates with an impaired NK cell activity (cytotoxicity, proliferation, and cytokine production) against PD-L^pos^ tumor cells that can be partially restored by mAb-mediated disruption of PD-1/PD-L interaction (41, 62, 69) (Figure 1).

This is an important detail because we know how *in vivo* the use of anti-PD-1 or anti-PD-L mAbs may generate beneficial effects toward the anti-tumor response mediated by T lymphocytes, but evidently also from NK cells.

Therefore, when we talk about tumor and NK cells we should not consider the recognition of HLA by the main inhibitory checkpoints expressed by NK cells, i.e., KIR or NKG2A, as the only system that plays a fundamental role in the control of tumor transformation, but we should also consider a possible participation of PD-1 in this system. In fact, simultaneous expression of different inhibitory checkpoints could provide multiple levels of suppression to anti-tumor responses of NK cells.

Now, several data suggest that NK cells are potential PD-1 blockade responders and that NK cell removal abrogates the anti-tumor efficacy of this immunotherapy (69).

Furthermore, PD-1 expression on NK cells may correlates with poor prognosis in different type of cancers (70). These findings strongly suggest a possible role for NK cells in immunotherapeutic strategies targeting the PD-1/PD-L1 axis particularly against HLA-I deficient tumor cells, but, interestinlgy, NK responses were still important for controlling cancer development also in cancer models in which CD8^+^ T cells played a substantial role (69) (Figure 1).

Thus, the analysis of expression/coexpression and function of inhibitory checkpoints is extremely important in order to design innovative immunotherapeutic strategies.

In this context, clinical trials are presently undergoing in which anti-NKG2A (monalizumab) or anti-KIR (lirilumab) antibodies are used as a combotherapy with anti PD-1 (nivolumab) for various type of solid tumors in order to obtain a complete reconstitution of anti-tumor NK cell citolytic activity (71).

These innovative approaches have a particular relevance especially if we think that tumor infiltrating T cells may express PD-1 but also KIR and/or NKG2A. Thus, the combined blockade of different checkpoints may simultaneously activate both innate and adaptive immune responses.

Interestingly, recent data indicate that PD-1 is also expressed by and may regulate both ILC2s and ILC3s, and that mAb-mediated blocking of PD-1 restored their effector functions. Since ILCs play a critical role in different inflammatory conditions, including tumors, these cells may represent interesting targets for immunotherapy (52, 72, 73) (Figure 1). Novel immunotherapeutic approaches could be based on the use of microRNA. In this context, it has been recently shown that the hsa-miR-146a-5p may negatively regulate the surface expression of certain KIRs by mimicking a “missing self” condition and, as a consequence, by improving the NK cell mediated cytotoxicity (74). Moreover, recent studies have provided novel evidence that miR-148a-3p and miR-873 negatively regulate tumor cell PD-L1 expression (75, 76). Thus, these regulatory miRNA/targets axes might serve as an additional tool in tumor therapy.

## Concluding Remarks

Tumor development often induces a suppressive microenvironment hampering cytotoxic lymphocytes effector-functions thus promoting tumor progression. T and NK cells result powerless just when we need them more. One of the main escape mechanisms by which tumor turn off our defense is the exploitment of immune checkpoints pathway. Restoring and harnessing immune cells to cure cancer represents an attractive challenge for scientists. In the 90s, Alessandro Moretta discovered the first NK cell immune checkpoints: KIRs and NKG2A. Soon after, Innate Pharma generated the first two therapeutic immune checkpoint inhibitors: lirilumab, targeting KIR, and monalizumab, targeting NKG2A. This was the beginning of a revolution. In the same years, Tasuku Honjo and Jim Allison discovered that the reactivation of the immune system by blocking two major immune checkpoints, CTLA-4 and PD-1, could represent a practice-changing approach in oncology.

Honjo and Allison received the Nobel Prize in Medicine 2018 “for their discovery of cancer therapy by inhibition of negative immune regulation.” During his speech at the Nobel Banquet, Honjo stated: “As a result, Jim and I have experienced many occasions that have made us feel well-rewarded, such as meeting cancer patients who say their lives were saved by our therapies.” He also added: “Jim and I both know that the development of our discovery is just beginning. We encourage many more scientists to join us in our efforts to keep improving cancer immunotherapy.”

Alessandro realized that immunotherapy with anti-PD-1 mAbs can be also useful for reactivating NK cells against the tumor in particular in the case of T-mediated tumor resistance (HLA-I^neg^ tumor cells). This intuition was confirmed by the identification of the PD-1 receptor on a subset of NK cells by his group. This subset is increased in the tumor microenvironment, as also shown by different research groups around the world (41, 62, 66).

The very recent discovery that PD-1 is also expressed on other groups of ILCs, including ILC2s and ILC3s, opens up new scenarios (52, 72, 73).

Once again Alessandro Moretta gave a fundamental contribution to the field of NK cell biology and to the clinical use of NK cell immune checkpoints. Initially, with the discovery of the first inhibitory checkpoints, then with the discovery of the various NK cell activating receptors (77), here not discussed, and finally, with the identification of the PD-1 immune checkpoint expression on NK cells. This latest discovery by Alessandro was certainly one of his most important contributions, and we collaborators, but above all friends, know what the discovery of this molecule on his beloved NK cells and its clinical implications has meant to him. Still today, all Alessandro's discoveries represent important bases for understanding how to best use NK cells in cancer therapies (Figure 1).

Thank you Alessandro for allowing us in your life, for supporting of us when we needed it most, for making us all always feel special and, most of all, for teaching us how to move forward with courage, simplicity and dignity.

Thank you Alessandro for having taught us how to do science, day by day, with scientific strictness, together with modesty. Thank you for having instilled in us your curiosity, and infinite passion for Science. Being afraid doesn't make any sense.

We miss you every moment in our life!

## Author Contributions

All authors listed have made a substantial, direct and intellectual contribution to the work, and approved it for publication.

### Conflict of Interest Statement

The authors declare that the research was conducted in the absence of any commercial or financial relationships that could be construed as a potential conflict of interest.
